# Characteristics and community stabilities of the resource islands formed by *Salix paraplesia* shrubs in the mobile sandy land of the upper reaches of the Yellow River in Sichuan Province

**DOI:** 10.1016/j.heliyon.2024.e40488

**Published:** 2024-11-16

**Authors:** Li He, Miaomiao Su, Dechao Chen, Houyuan Zeng, Xuemei Huang, Honglin Li, Shilei Wu, Hang Song, Xue Jiang, Kejun Wu, Jingyu Yang, Wuxian Yan, Dongzhou Deng

**Affiliations:** a Sichuan Academy of Forestry, Chengdu, 610081, China; b Tibet Autonomous Region Institute of Science and Technology Information, Tibet, 850000, China; c Sichuan Provincial Forestry and Grassland Key Laboratory of Combating Desertilicatlon, Chengdu, 610081, China; d College of Life Science and Biotechnology, Mianyang Normal University, Mianyang, 621000, China; e Engineering Research Center for Forest and Grassland Disaster Prevention and Reduction, Mianyang Normal University, Mianyang, 621000, China

**Keywords:** *Salix paraplesia*, Resource Island, Species diversity, Community stability, Mobile sandy land

## Abstract

This study comprehensively analyzes the physical and chemical properties of soil across different layers under *Salix paraplesia* shrubs, comparing these properties inside and outside the shrub canopies. It also examines species diversity and community stability in these areas, discussing the impact of soil from resource islands at different formation stages on vegetation restoration. Focusing on *Salix paraplesia* shrubs over varying restoration periods (45 years, 25 years, and 13 years), with unrepaired mobile sandy land serving as the control, the results are as follows: (1) As vegetation restoration progresses, the content of soil organic matter (SOM) and alkali-hydrolyzable nitrogen (AN) significantly increase. After 45 years of restoration, SOM and AN contents are 250.08 % and 442.43 % higher than in the control. SOM, AN, and total phosphorus (TP) contents are greater under the shrubs than outside the canopies, indicating pronounced fertilizer accumulation. (2) The number of plant species increases during the restoration process, with 18 more species present after 45 years compared to the control. Community diversity is highest after 13 years of restoration, with greater overall diversity outside the canopies than under the shrubs. These differences decrease as restoration progresses. (3) The stability of *Salix paraplesia* shrub communities is higher outside the canopies compared to under the shrubs. As restoration proceeds, stability differences between these areas diminish. The Euclidean distances for community stability outside and under the canopies are 13.97 and 14.68, respectively, after 45 years, indicating relative stability. (4) In the early stages of restoration, soil carbon (C) and nitrogen (N) significantly impact species diversity, while in the later stages, phosphorus (P) content becomes more limiting. Resource islands enhance plant community stability and vegetation restoration, playing a crucial role in ecological protection and vegetation restoration in the alpine sandy land of the Yellow River's upper reaches.

## 1 Introduction

Resource Islands, also known as Fertile Islands, were first conceptualized by Klemmedson in 1985 [1]. This phenomenon has been observed in various regions and under different types of shrubs [2,3]. Resource islands under shrubs highlight the significant heterogeneity of soil resources [4]. It is widely accepted that their formation is influenced by factors such as shrub species, crown width, height, and the presence of biological soil crusts. The "fertile island" effect is closely tied to plant growth stages and habitat conditions [5,6]. Current research on resource islands primarily focuses on their formation mechanisms, descriptive phenomena, and impacts on ecological processes in arid and semi-arid regions [[7], [8], [9], [10]]. Liu et al. [11] identified fertile island effects in tamarix shrub sand drifts. Studies show that in extremely degraded areas like mobile sandy lands, artificially planted shrubs adapted to harsh environments can foster the formation of "fertile islands" [12], illustrating the positive feedback between resource islands and shrub communities. Liu and Guo [13] indicated that shrub resource islands would not effectively promote the growth and resource utility of all the indigenous tree species and shorten the reforestation course in subtropical degraded shrubland in South China. Therefore, it can be inferred that there must be a correlation between resource islands and plant communities.

Stability is a comprehensive characteristic of plant community structures and functions, encompassing resistance and resilience stability [14,15]. Key factors affecting community stability include management practices [16], functional diversity and redundancy [17], types and degrees of disturbance [18], and plant functional groups [19]. There is no universally accepted method for measuring plant community stability. Common methods include the M Godron stability measurement, succession comparison, comprehensive evaluation, and coefficient of variation methods. Identifying suitable methods and standards for assessing post-restoration community stability in sandy lands is crucial for their management and restoration. The M Godron stability measurement is known for its simplicity and standardization. Currently, an improved version of this method [20] is widely used to measure community stability. Planting shrubs is a common strategy for promoting vegetation restoration in sandy lands. Research indicates that shrub planting in mobile sandy land can reduce soil sand content, increase the presence of extremely fine sands and adhering powders, and promote surface vegetation restoration [7,21]. Historically, scholars have focused more on soil conditions or vegetation characteristics of sand-fixing plants in the upper reaches of the Yellow River, often using singular evaluation factors. Quantitative analyses and research on the survival stability of dominant plants in the region are still lacking.

The upper reaches of the Yellow River in Sichuan Province, known as the "reservoir" of the Yellow River, are a crucial component of China's water ecosystem. This region is a significant national ecological function area with a strategic role in the ecological security of the Yellow River basin. However, the regional ecosystem is notably fragile. Monitoring results indicate that the area of desertified land in this region is nearly 7 million hectares, posing a major ecological challenge that threatens the long-term stability of both the upper reaches of the Yellow River in Sichuan and the nation. *Salix paradesia* is a shrub from the family Salicaceae, renowned for its adaptability to arid and infertile sandy soils in the alpine regions of northwest Sichuan [22]. It plays a pivotal role in sandy land restoration. Prior studies have primarily focused on the causes of regional desertification [23,24], vegetation and soil characteristics, and their response to vegetation restoration efforts [[25], [26], [27]]. However, there has been limited investigation into the effects of resource islands and community stability formed by shrubs in the upper reaches of the Yellow River. This study focuses on *Salix paraplesia* shrubs in the mobile sandy land of the upper reaches of the Yellow River in Sichuan Province. We examined the physical and chemical properties of soil across different layers, species diversity, and stability both inside and outside the shrub canopies. Our objective was to evaluate the stability of *Salix paraplesia* communities and to uncover the changing characteristics and evolutionary processes of the "fertile island" effect generated by these shrubs. Additionally, we explored the impact of resource island soils, formed at different times, on vegetation restoration. This study aims to provide a scientific basis and theoretical support for the restoration and scientific management of vegetation in the sandy lands of the upper Yellow River.

## 2 Methodology and data

### 2.1 Overview of the study area

The research area is located in Zoige, a pilot county for desertification control in the alpine sandy area of northwest Sichuan. Zoige is situated at the northeast edge of the Qinghai-Tibet Plateau in the northern part of Sichuan Province, with geographical coordinates between 102° 08 ′ −103° 39 ′ E and 32° 56 ′ −34° 19 ′ N. This region experiences a plateau continental climate, with average annual precipitation ranging from 650 mm to 750 mm and a relative humidity of approximately 70 %. The annual average temperature is about 0.7 °C, with extremes reaching 24.6 °C and −33.7 °C. The area receives an annual sunshine duration of 2573 h. The predominant monsoon winds are northeasterly and southwesterly, with an average wind speed of 2.4 m s^−1^ and a maximum wind speed of 40 m s^−1^ [27].

The study area is characterized by typical alpine meadows, with vegetation primarily comprising xerophytic and halophytic shrubs or herbs. This area represents a typical steppe ecosystem. The dominant shrub species in the community is *Salix paraplesia*, and the main herbaceous plants include species from the genera *Carex*, *Cyperus*, *Poa*, *Potentilla*, *Ligularia*, and *Saussurea*.

### 2.2 Sample plot setting and investigation

**Fig. 1:**
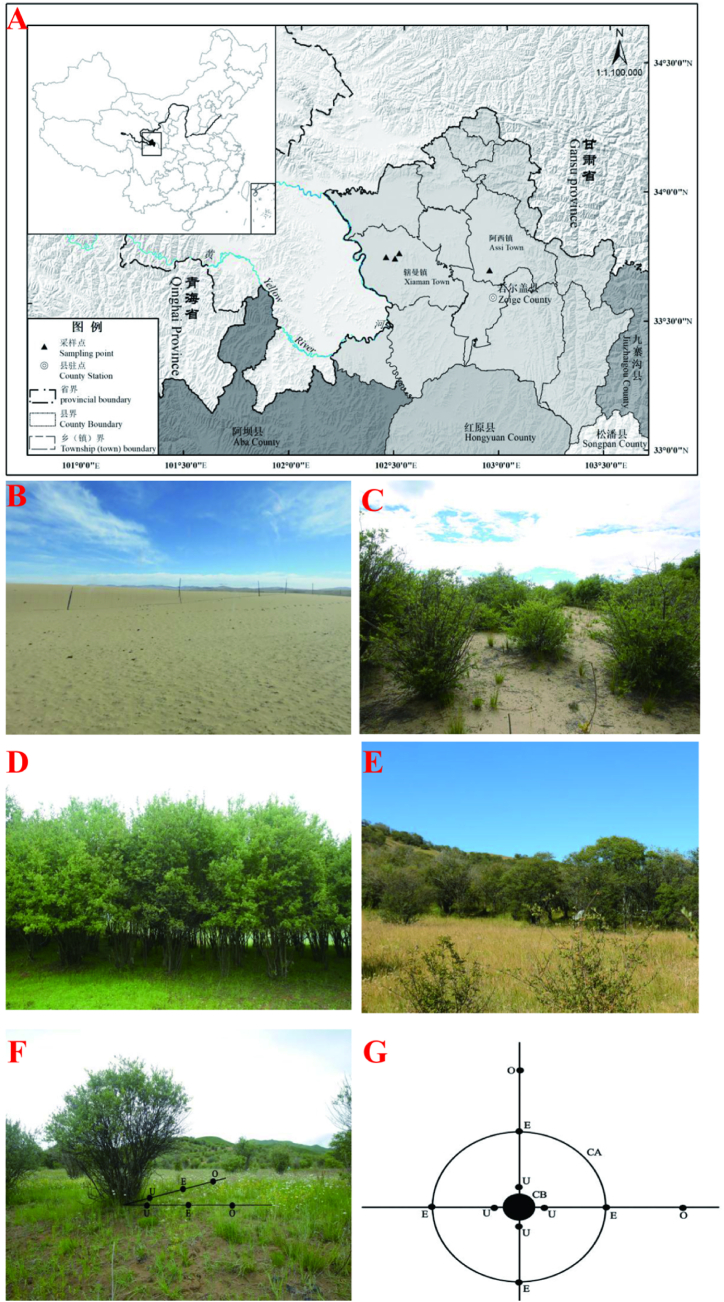
**The schematic diagram of Salix paraplesia shrubs and sampling points.** A is a sampling point map, B is the mobile sandy land, C is the 13 years of the *Salix paraplesia* community, D is the 25 years of the *Salix paraplesia* community, E is the 45 years of the *Salix paraplesia* community, F and G are the distribution map of sample points. CA: vertical projection of S. paraplesia crown; CB: vertical projection Vertical projection of S. paraplesia crown base.O: Extracoronal sampling points Sampling point outside of crown; E: sampling point edge of canopies; U: sampling point under the crown.

This study employs the space-for-time substitution method, which uses spatial differences to infer temporal changes. In 2021, in the mobile sandy land areas of Xiaman village, Zoige County, we selected *Salix paraplesia* communities that had undergone restoration for 45, 25, and 13 years as our research sample plots (Table 1, Fig. 1A–E). In each plot, we randomly established more than three 5 m × 5 m quadrats of *Salix paraplesia* communities, resulting in a total of 12 quadrats. We investigated community characteristics, recording species names, heights, branch numbers, coverage, and crown widths of the shrubs in each quadrat. Additionally, quadrat surveys (0.5 m × 0.5 m) were conducted in four directions (under and outside the canopy of *Salix paraplesia* shrubs) to record data on species numbers, plant heights, coverage, and abundance. Plant specimens were collected for further identification in the laboratory.

**Table 1:** Basic information of sample plots.

Recovery time	Location	Longitude & Latitude	Elevation(m)	Restoring measures
0(CK)	Axi vill., Zoige	E102°56′22.77″ N33°41′7.91″	3455	Willow sand barrier+planting irrigation+grass planting+fertilization
13	Xiaman vill., Zoige (implemented in 2008)	E102°30′13.87″ N33°44′36.57″	3434	
25	Xiaman vill., Zoige (implemented in 1996)	E102°29′2.89″ N33°43′20.29″	3466	
45	Xiaman vill., Zoige implemented in 1976	E102°26′24.41″ N33°43′37.40″	3452

### 2.3 Sample collection

After surveying the vegetation characteristics in each sample plot, we collected soil samples using a 3.5 cm diameter soil drill. Samples were taken to a depth of 60 cm in three layers (0–20 cm, 20–40 cm, 40–60 cm) under the *Salix paraplesia* canopy, at the canopy edge, and outside the canopy, following the directions of east, south, west, and north. Samples from the same depth were combined to form a composite sample. Three well-grown *Salix paraplesia* shrubs were selected for each sample, resulting in a total of 90 soil samples. The samples were homogenized, and plant roots and large stones were removed. We then collected 0.5 kg of each sample in valve bags for laboratory analysis. Soil moisture content was measured immediately, and the remaining samples were air-dried to determine soil pH, Soil organic matter (SOM),Alkali-hydrolyzable nitrogen, Total phosphorus (TP).

Soil pH values were determined using the electrode potential method (1:2.5 soil/water extracts). SOM was measured using the potassium dichromate oxidation-colorimetry method [11]. Alkali-hydrolyzable nitrogen content in the soil was determined using the method of Lu [28]. Total TPcontent in the soil was measured using the molybdenum antimony scandium colorimetry method [29].

### 2.4 Analytical methods

#### 2.4.1 Species diversity analysis

The species diversity index reflects the complexity and organizational level of community structure and function, providing a systematic and clear overview of community ecological habits. The diversity calculations were performed using the following formulas:

Importance value of plant species, refer to Formula (1):

(1) Iv=(Relativeheight+Relativecoverage+Relativedensity)/3

**Measurement of α diversity:** Community species richness, Shannon-Wiener diversity index, Simpson's dominance diversity index, and Pielou's evenness index were used. The calculation formulas are based on Chu et al. [30] and He et al. [27].

Margalef's richness index, refer to Formula (2):

(2) R=(S−1)/lnN

Simpson's dominance index, refer to Formula (3):

(3) $$D=1－\sum_{i=1}^{s}{P_{i}}^{2}$$

Shannon-Wiener diversity index, refer to Formula (4):

(4) $$H=－\sum_{i=1}^{s}P_{i}\;\ln\;P_{i}$$

Pielou's evenness index, refer to Formula (5):

(5) $$E=\left(－\sum_{i=1}^{s}P_{i}\;\ln\;P_{i}\right)/\text{lnS}$$

In these formulas, *Ni* was the number of individuals (species importance value) of species *i*; *N* was the total number of individuals (species importance value) of all species; *Pi* referred to the ratio of *Ni* to *N*, i.e. Pi = Ni/N, i = 1, 2, 3, …, S; *S* was the total number of species.

#### 2.4.2 Community stability analysis

Community stability was assessed using the method of M. Godron [31]. This involves calculating the number and frequency of all species in the plant community and plotting these on a scatter diagram, correlating the cumulative percentage of plant species with the cumulative relative frequency (Fig. 1F and G). A smooth curve is drawn through each point, with a straight line connecting the 100 points on both coordinate axes. The intersection of this straight line with the curve represents the stability point. According to this method, a community is considered stable when the ratio of the cumulative percentage of plant species to the cumulative relative frequency is close to 20/80. If the intersection point's distance from the stability point (20,80) ≤ $\sqrt{10^{2}+10^{2}}=14.142$, the community is deemed stable. Otherwise, it is considered unstable [27,32]. In this study, we improved Godron's stability measurement method by replacing species frequency with coverage.

#### 2.4.3 Data analysis

SPSS Statistics (version 16.0, Chicago, USA) was used to describe and analyze various indicators. One-way ANOVA and LSD tests were conducted to compare species diversity and soil nutrient indicators between different parts of the same recovery years and between the same parts of different years. Figures were created using Origin 8.5 (Origin Lab Corporation, MA, USA), R software (version 4.2.2), and Adobe Illustrator 22.0.

## 3 Results and analysis

### 3.1 Characteristics of resource island formed by *Salix paraplesia* shrubs

**Fig. 2:**
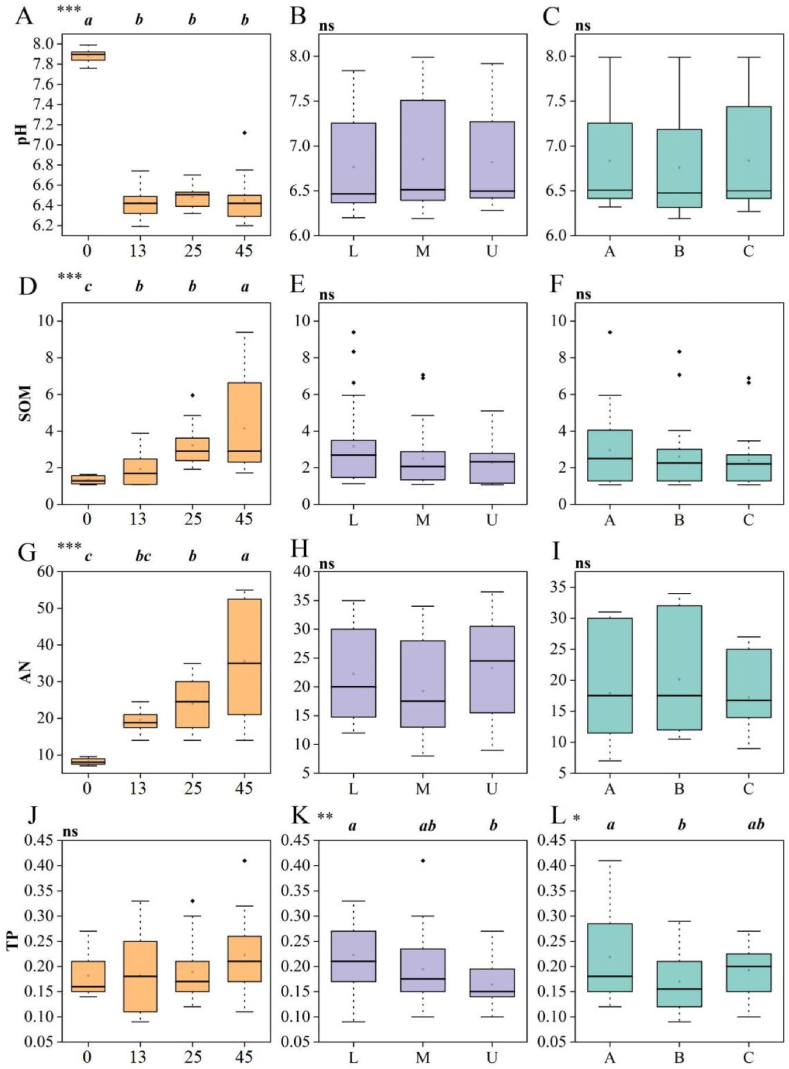
**Soil physical and chemical properties under different restoration time, soil depth, and position of *Salix paraplesia* shrubs.** A, D, G, and J represented 0 years, 13 years, 25 years, 45 years of recovery, respectively. B, E, H, and K represented 0–20 cm (L), 20–40 cm (M), 40–60 cm (U) soil layers, respectively. C, F, I, and L represented under the crown (G1), at the edge of the crown edge (G2), and outside the crown (G3), respectively. ∗∗∗ indicated that the differences between different recovery times is significant (*p* < 0.001), and the same lowercase letter indicated that the differences among the data were not significant.

The temporal and spatial variations of soil pH, soil organic matter (SOM), alkali-hydrolyzable nitrogen (AN), and total phosphorus (TP) across different restoration years are illustrated in Fig. 2A–L and Table 2. During vegetation restoration, soil pH values for each restoration year were significantly lower than the control. After 45 years of restoration, SOM and AN contents were 250.08 % and 442.43 % higher than the control, respectively. However, there were no significant differences in soil TP content among the different restoration years. Vertically, only the TP content in the 0–20 cm soil layer was significantly higher than that in the 0–60 cm soil layer. SOM was primarily distributed in the 0–20 cm shallow soil layer, with no significant differences among different soil layers. AN content also showed no significant change among the different soil layers. Comparing the indices under the crown, at the edge of the crown, and outside the crown revealed that only TP content under the crown was significantly higher than at the edge of the crown (*P* < 0.05). While SOM, AN, and TP contents were higher under the crown compared to outside, the differences were not statistically significant.

**Table 2:** Interaction effects among different restoration years, different soil depths and different parts of the plant.

Indexes	Factor	Df	F	P
pH	Restoration years	3.000	480.495	0.000∗∗∗
	Soil depth	2.000	2.343	0.110
	Parts	2.000	2.504	0.096
	Recovery years: soil depth	6.000	0.852	0.539
	Recovery years: parts	6.000	0.836	0.550
	Soil depth: parts	4.000	0.491	0.742
	Recovery years: soil depth: Parts	12.000	1.271	0.277
AN	Restoration years	3.000	10.683	0.000∗∗∗
	Soil depth	2.000	0.434	0.651
	Parts	2.000	0.160	0.852
	Recovery years: soil depth	6.000	0.839	0.548
	Recovery years: parts	6.000	0.828	0.556
	Soil depth: parts	4.000	1.247	0.309
	Recovery years: soil depth: Parts	12.000	1.381	0.220
TP	Restoration years	3.000	5.533	0.175
	Soil depth	2.000	55.350	0.006∗∗
	Parts	2.000	11.138	0.034∗
	Recovery years: soil depth	6.000	2.400	0.047∗∗
	Recovery years: parts	6.000	1.906	0.107
	Soil depth: parts	4.000	3.523	0.016∗∗
	Recovery years: soil depth: Parts	12.000	1.013	0.457
SOM	Restoration years	3.000	51.934	0.000∗∗∗
	Soil depth	2.000	18.845	0.619
	Parts	2.000	4.663	0.263
	Recovery years: soil depth	6.000	7.192	0.000∗∗∗
	Recovery years: parts	6.000	0.896	0.506
	Soil depth: parts	4.000	0.602	0.663
	Recovery years: soil depth: Parts	12.000	1.683	0.103

Notes: ∗∗∗p < 0.001, ∗∗p < 0.01, ∗p < 0.05.

### 3.2 Species diversity of *Salix paraplesia* shrub community

Table 3 shows significant changes in the number of plant species in the mobile sandy land since the implementation of vegetation restoration measures. The number of species increased from 3 to 21, with annual herb species rising from 0 to 4 and herbaceous perennial species increasing by 14. After 45 years of restoration, the mobile sandy land was predominantly occupied by *Leymus secalinus*, *Carex moorcroftii*, *Elymus nutans*, *Poa pratensis*, and *Kobresia robusta* communities. Of the 21 species, 17 were perennial herbs (80.95 %) and 4 were annual herbs (19.05 %).

**Table 3:** Changes of important values of dominant species during vegetation restoration.

Main species	Restoration years			
	0	13	25	45
*Carex moorcroftii*	0.50	0.34	0.20	0.21
*Leymus secalinus*	0.22	0.17	0.12	0.14
*Agropyron cristatum*		0.12	0.11	
*Poa pratensis*			0.17	0.22
*Elymus nutan*		0.35	0.24	0.45
*Kobresia robusta*		0.16	0.12	0.40
*Heracleum millefolium*		0.38	0.15	0.09
*Hypecoum leptocarpum*		0.12	0.06	0.07
*Potentilla bifurca*	0.07			
*Vicia cracca*		0.45	0.09	0.05
*Artemisia desertorum*		0.11	0.16	
*Corispermum declinatum*		0.07		
*Oxytropis falcata*		0.45	0.08	0.15
*Ajania tenuifolia*			0.04	
*Carum carvi*		0.16		0.07
*Ligularia virgaurea*		0.13		0.12
*Aconitum gymnandrum*				0.05

In the untreated mobile sandy land, *C. moorcroftii* and *L. secalinus* were the dominant species with important values of 0.50 and 0.22, respectively. As recovery time extended, species numbers increased, and the important values of *C. moorcroftii* and *L. secalinus* decreased, reducing their dominance. After 45 years of restoration, the important values of *C. moorcroftii* and *L. secalinus* decreased to 0.21 and 0.14, respectively, while the important values of *E. nutans* and *K. robusta* increased to 0.45 and 0.40, respectively, with *E. nutans* becoming a significant community species.

**Fig. 3:**
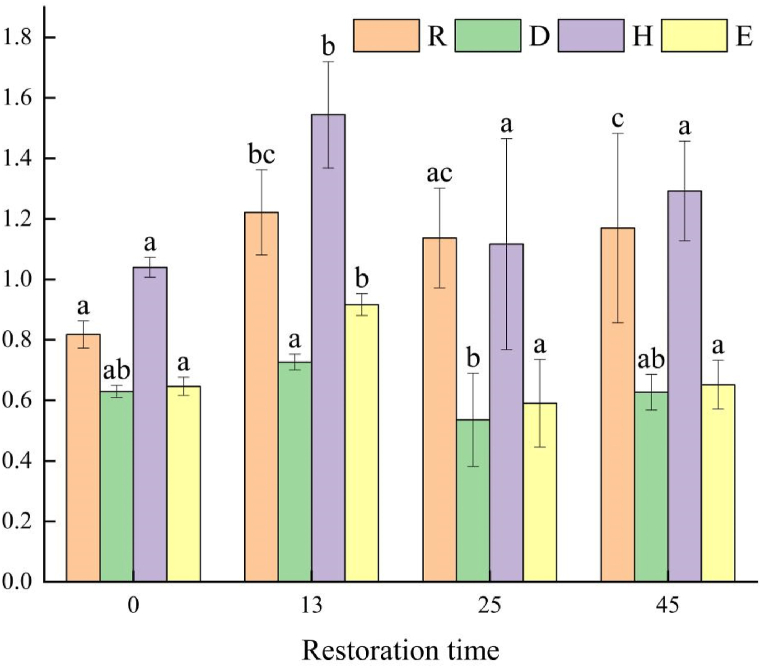
Diversity indexes of *Salix paraplesia* shrub community under different recovery time.

**Fig. 4:**
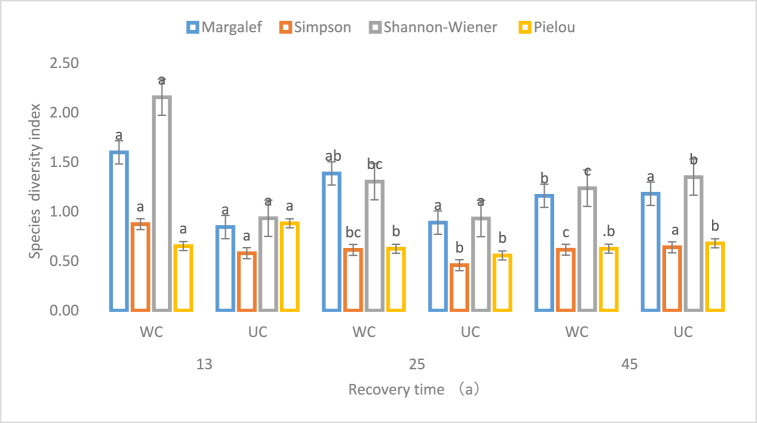
**The diversity index under and outside the crown of *Salix paraplesia* willow shrubs.** Different lowercase letters indicated that there were significant differences among the diversity index under and outside the crown under different recovery years (P < 0.05). WC:without the crown; UC:under the crown.

The diversity measurements of *Salix paraplesia* shrub communities at different restoration years (Fig. 3 and Table 4) indicated that plant community diversity indices initially increased, then decreased, and increased again over time. However, only the Richness index, Shannon-Wiener index, and Pielou index of the 13-year restoration plots were 49.37 %, 48.50 % and 41.76 % significantly higher than those of the control. Fig. 4 shows that the Simpson index, Shannon-Wiener index, and Pielou index were significantly higher in the plant community after 13 years of restoration compared to those after 25 and 45 years outside the crown, Conversely, the Shannon-Wiener index after 45 years was significantly higher than those after 25 and 13 years under the crown. It was 45.02 % and 44.66 % higher than those after 25 years and 13 years.The diversity index of the 13-year and 25-year plots was larger outside the crown than under it, with no significant differences in the diversity index between under and outside the crown of *Salix paraplesia* shrubs after 45 years of restoration.

**Table 4:** Interaction effects amongdifferent recovery years and different parts of the plant.

Indexes	Factor	Df	F	P
R	Restoration years	3	6.708	0.101
	Parts	1	22.468	0.006∗∗
	Restoration years: parts	3	7.4836	0.002∗∗
D	Restoration years	3	4.026	0.084
	Parts	1	7.497	0.044∗
	Restoration years: parts	3	3.205	0.048∗
H	Restoration years	3	6.072	0.147
	Parts	1	17.442	0.021∗
	Restoration years: parts	3	10.711	0.000∗∗∗
E	Restoration years	3	15.484	0.000∗∗∗
	Parts	1	0.271	0.756
	Restoration years: parts	3	0.399	0.756

Notes: ∗∗∗p < 0.001, ∗∗p < 0.01, ∗p < 0.05; R. Margalef’s richness index; D. Simpson's dominance index; H. Shannon-Wiener diversity index; E. Pielou's evenness index, same below.

### 3.3 The stability of *Salix paraplesia* shrub community

The stability of the *Salix paraplesia* shrub community was evaluated by simulating vegetation conditions under and outside the crown across different restoration years using smooth curves. A good fit for the plant community stability curve equation (with a determination coefficient greater than 0.90) indicates a reliable simulation. To facilitate visual interpretation, the distance between the intersection point coordinates and the equilibrium point coordinates was expressed as the Euclidean distance (Table 5).

**Table 5:** Stability of plant community under and outside *Salix paraplesia* shrubs under different recovery years.

Restoration years	Positions	Fitting curves	R^2^	Intersection coordinates	Euclidean distance	Result
0	–	y = 0.833x + 16.66	–	45.48, 54.52	36.03	Instability
13	Under the crown	y = 0.02x^2^+0.5x+10-14	0.996∗∗	42.54, 57.06	31.88	Instability
	Outside the crown	y = −0.010x^2^ + 1.857x +14.51	0.983∗∗	33.95, 66.05	19.73	Instability
25	Under the crown	y = −0.010x^2^ + 1.82x + 20.09	0.937∗∗	31.96, 68.04	16.91	Instability
	Outside the crown	y = −0.014x^2^ + 2.287x + 12.54	0.975∗∗	30.59, 69.41	14.98	Instability
45	Under the crown	y = −0.012x^2^ + 1.989x + 20.27	0.987∗∗	30.38, 69.62	14.68	Instability
	Outside the crown	y = −0.010x^2^ +1.772x + 26.09	0.985∗∗	29.88, 70.12	13.97	Instability

The Euclidean distance between the intersection coordinate and the stable point serves as an index to assess community stability [31]. As shown in Table 5, stability was consistently higher outside the crown compared to under the crown for each restoration year, with the most pronounced difference observed in the 13-year restoration plots. The coordinates outside the crown were (33.95, 66.05), with a related Euclidean distance of 19.73, which was less than the Euclidean distance within the crown (31.88). As restoration progressed, the stability difference between under and outside the crown decreased. Overall, plant community stability gradually increased and tended to stabilize over time. After 45 years of restoration, the Euclidean distances outside and under the crown were 13.97 and 14.68, respectively.

### 3.4 Effects of soil physical and chemical properties on species diversity

**Fig. 5:**
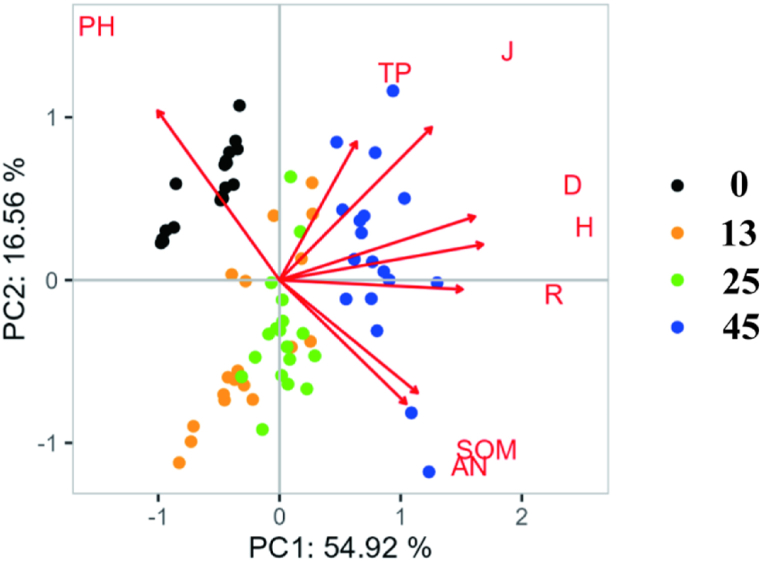
Principal component analysis of soil physical and chemical properties and species diversity of willow shrublan.

Principal component analysis (PCA) of eight indices related to species diversity and soil physical and chemical properties across different restoration years was conducted. The contribution rate of the first principal component (PC1) was 53.06 %, while the second principal component (PC2) contributed 15.91 % (Fig. 5). Together, the cumulative contribution rate of the principal components reached 68.97 %. The diversity index was positively correlated with SOM, AN, and TP, and negatively correlated with soil pH. The influence of these factors was ranked based on the length of the projection to the abscissa as follows: SOM > AN > TP. In the early stages of restoration, soil carbon (C) and nitrogen (N) had a significant impact on species diversity. However, in the later stages of restoration, soil phosphorus (P) content became the limiting factor.

## 4 Results and discussion

### 4.1 Characteristics of resource island formed by *Salix paraplesia* shrubs

In desertification ecosystems, establishing sand-fixing vegetation is crucial for restoring desertified land [33]. The survival of shrubs in mobile sandy land promotes nutrient cycling and improves soil physical and chemical properties [33,34]. The spatial distribution differences in soil pH primarily appear at varying soil depths and between areas under and outside the shrubs over different restoration years, highlighting the significant effects of shrubs on soil properties. Soil pH values for each restoration year were significantly lower than the control, with pH values under the shrubs being lower than those outside. These findings are consistent with Su et al. [35], indicating that the extensive secretion of organic acids and the release of CO_2_ from litter, roots, and microorganisms can lead to decreased pH levels [35,36]. These results demonstrate the positive impact of sand-fixing shrubs on soil pH.

Our study showed that soil nutrient content decreases with distance from the main stem of *Salix paraplesia*. SOM, AN, and TP contents in the 0–20 cm soil layer under *Salix paraplesia* shrubs were higher than those outside the crown, aligning with the findings of Yin et al. [37] and Zhao et al. [38]. This phenomenon is linked to the establishment of shrubs resistant to wind and sand, litter decomposition, and root regeneration [39]. Numerous studies have shown that shrubs effectively prevent soil wind erosion [40,41]. The establishment of *Caragana microphylla* traps wind-blown fine materials and dust rich in nutrients, which then deposit in the surface soils under their canopies [42]. These nutrients accumulate in the soil, enriched by root litter more so than herbaceous plants. Litter decomposition increases soil organic matter and nutrient content [43], with N released by litter decomposition enhancing soil N levels [35,44], resulting in better soil conditions under the shrub layer compared to surrounding areas [45]. As sand-fixation continues over the years, the rapid growth and death of shrubs provide essential inputs of C, N, and other nutrients [46]. During their growth, shrubs reduce surface runoff and intercept nutrients, allowing soil to chelate C, N, and P, thereby increasing their content [47]. The root structure of shrubs plays a critical role in improving soil nutrients. More roots increase contact area with soil and enhance sand fixation capacity [44], consistent with other research in arid and semiarid regions [4,35,44,48]. Our results indicate higher levels of SOM, AN, and TP under shrub canopies compared to areas outside the shrubs, forming a clear resource island, suggesting that soil nutrient initiation occurs under the shrub crown. Artificially established vegetation significantly improves soil properties [35,44], but the accumulation of C, N, and P nutrients in the soil surface is significantly influenced by the duration of vegetation restoration [48,49].

### 4.2 Differences in species composition and the diversity of community with different restoration years

Vegetation restoration in degraded ecosystems essentially involves the succession of plant communities [50]. Along the gradient of vegetation restoration, dynamic changes in species composition and vegetation community succession in sandy land reflect the response of the community environment to these changes [51]. Numerous studies have demonstrated that vegetation restoration measures in the sandy lands of the alpine region of northwest Sichuan improve soil structure and fertility, subsequently affecting the composition of above-ground vegetation [21,52,53]. During vegetation restoration, not all species play equal roles in the community or ecosystem. The ecological functions of dominant and key species are irreplaceable [50,51,54]. In ecologically fragile areas, such as arid desertified regions, ecosystem function maintenance depends significantly on major populations [51]. Our study revealed that with extended restoration time, species numbers increased significantly, and plant community composition became more complex. The appearance of plant communities also showed considerable changes. Species and numbers of plants, particularly those in the families Gramineae and Cyperaceae, increased, while the importance values of *Carex qinghaiensis* and *Leymus secalinus* decreased. This is consistent with similar research findings [50,51,55,56] and can be attributed to the emergence of plants from families such as Ranunculaceae and Geraniaceae (*Ranunculus tanguticus*, *Anemone rivularis*, *Geranium nepalense*) in later stages of vegetation restoration. These changes indicate that after implementing vegetation restoration measures in mobile sandy land, the ecosystem has entered a stage of increasing plant species diversity, signaling the beginning of recovery and succession. During vegetation restoration, the number of perennial herbaceous plants increased significantly, with only four species of annual herbaceous plants remaining after 45 years of restoration. This species composition reflects the ecosystem's adaptability to extreme environments and suggests that perennial plants play a constructive role in the early stages, while annual plants act as pioneer species in specific niches.

Species composition is a fundamental characteristic of plant communities, indicating changes in the relationship between the community and the surrounding environment. It reflects the degree of ecosystem degradation and restoration [57]. Our study showed that the diversity of vegetation communities in the mobile sandy land initially increased, then decreased, and increased again over the restoration period. With extended recovery time, plants from families Ranunculaceae, Asteraceae, and Leguminosae gradually sprouted and grew rapidly. In the mobile sandy land of the upper Yellow River, due to extremely poor soil and the effects of wind and sand, only a few pioneer herbaceous plants could survive and reproduce [25]. The native vegetation of untreated mobile sandy land serves as the starting point for vegetation restoration in this type of sandy land, with the lowest species diversity. This finding aligns with similar research results from the Qilian Mountains [50] and Horqin Sandy Land [51]. When the recovery time reached 13 years, the diversity index was at its highest, related to a significant increase in herbaceous perennials, followed by a decrease. After 25 years of recovery, diversity increased slowly, likely because the mobile sand had stabilized, improving competitiveness among species and allowing for the invasion of other species, which increased community diversity.

As a shelter plant, *Salix paraplesia* significantly impacts the growth of herbaceous plants under its canopy. The species diversity of herbaceous plants in the shrubs was greater outside the crown than under it during the 13 and 25-year recovery periods, consistent with the findings of Wang et al. [9]. After 45 years of restoration, differences between areas under and outside the crown were minimal, indicating that the composition and structure of the herbaceous plant community under the crown in the mobile sandy land tended to become uniform during vegetation restoration. Studies have shown that sandy shrublands can reduce wind speed, intercept atmospheric dust and organic litter [42,58], and dense branches can prevent or reduce herbivore feeding [59]. This allows non-pioneer herbaceous plants to be preserved under the shrubs in mobile sandy land, enabling normal growth and reproduction [60]. This protective effect of shrubs on lower herbaceous plants is crucial for maintaining the stability of sandy vegetation [60,61].

### 4.3 Differences in community stability among different restoration years

The stability of plant communities plays a crucial role in the sustainability of grassland ecosystems and is considered an important indicator of ecosystem health [62,63]. Exploring the stability of *Salix paraplesia* shrub communities at different recovery times involves understanding the effects of resource island soils of varying ages on community stability. In the early stages of growth, the characteristics of the formed fertile islands are not pronounced, and the recovery of herbaceous plants under the crown is the primary focus. As *Salix paraplesia* shrubs grow, the resource island characteristics become more evident, promoting the growth of herbaceous plants under the crown and gradually increasing community stability. Our study found that after 13 years of recovery, community stability was lower than that of the 25-year and 45-year communities, despite having the highest species diversity. This indicates that the community with the highest species diversity during vegetation restoration does not necessarily have the most stable community with the longest succession history [51]. The species diversity of the community with a 13-year succession period was the highest, representing an intermediate stage of community succession. Similar results were obtained by Zhang et al. [51] in the study of Horqin sandy land.

## 5 Conclusion

During the vegetation restoration of mobile sandy land in the upper reaches of the Yellow River in Sichuan, changes in soil nutrients, species diversity, and plant community stability are closely related to the recovery time. SOM and AN contents increased significantly with recovery time, enhancing the resource island effect. As restoration progressed, shrub community stability increased and tended to stabilize. Generally, the stability and species diversity of the community outside the crown were higher than those under the crown, with differences narrowing over time as vegetation restoration continued. Resource island soils effectively promote vegetation restoration, particularly benefiting under-crown herbaceous plants. Analysis of soil physical and chemical properties on species diversity revealed that SOM and AN significantly influenced species diversity in the early restoration stages, while soil TP content became limiting in the later stages. After ecological restoration of mobile sandy land, the *Salix paraplesia* shrub community formed a resource island, providing essential conditions for community restoration and succession, thereby enhancing plant community stability. It is recommended to apply organic fertilizers during the early stages of shrub restoration to promote species diversity. In the later stages, phosphorus fertilizers should be used to maintain the diversity and stability of the shrub community.

## CRediT authorship contribution statement

**Li He:** Writing – review & editing, Writing – original draft, Visualization, Validation, Supervision, Software, Resources, Project administration, Methodology, Investigation, Formal analysis, Data curation, Conceptualization. **Miaomiao Su:** Writing – review & editing, Writing – original draft, Visualization, Supervision, Software, Resources, Methodology, Investigation, Formal analysis, Data curation, Conceptualization. **Dechao Chen:** Writing – original draft, Visualization, Software, Resources, Project administration, Methodology, Investigation, Formal analysis, Data curation, Conceptualization. **Houyuan Zeng:** Writing – original draft, Visualization, Software, Methodology, Investigation, Formal analysis, Data curation, Conceptualization. **Xuemei Huang:** Visualization, Validation, Software, Resources, Methodology, Investigation, Formal analysis, Data curation. **Honglin Li:** Writing – original draft, Visualization, Validation, Methodology, Investigation, Data curation, Conceptualization. **Shilei Wu:** Writing – original draft, Visualization, Software, Methodology, Formal analysis, Data curation. **Hang Song:** Visualization, Validation, Software, Methodology, Investigation. **Xue Jiang:** Writing – original draft, Visualization, Software, Methodology. **Kejun Wu:** Writing – review & editing, Writing – original draft, Supervision, Formal analysis, Data curation. **Jingyu Yang:** Writing – review & editing, Writing – original draft, Validation, Data curation. **Wuxian Yan:** Writing – review & editing, Writing – original draft, Validation. **Dongzhou Deng:** Writing – review & editing, Writing – original draft, Visualization, Validation, Supervision, Software, Resources, Project administration, Methodology, Investigation, Funding acquisition, Formal analysis, Data curation, Conceptualization.

## Availability of data and materials

The data and materials can be obtained from the author on reasonable request.

## Funding

The research was financially supported by the Sichuan Provincial Science Basic Scientific Research Projects (No. 2021JBKY13), Science and Technology Innovation Project of Provincial Forestry and Grass Bureau (No. 510201202109731), Ecological and Security Key laboratory of Sichuan Province, China (ESP2201), and Sichuan Provincial Science and Technology Department Project (No. 2023NSFSC0750).

## Declaration of competing interest

The authors declare that they have no known competing financial interests or personal relationships that could have appeared to influence the work reported in this paper.
